# Enhancing the efficacy of immunotherapy using radiotherapy

**DOI:** 10.1002/cti2.1169

**Published:** 2020-09-10

**Authors:** Synat Keam, Suki Gill, Martin A Ebert, Anna K Nowak, Alistair M Cook

**Affiliations:** ^1^ National Centre for Asbestos Related Diseases Perth WA Australia; ^2^ School of Medicine The University of Western Australia Perth WA Australia; ^3^ Department of Radiation Oncology Sir Charles Gairdner Hospital Perth WA Australia; ^4^ School of Physics, Mathematics and Computing The University of Western Australia Perth WA Australia; ^5^ Department of Medical Oncology Sir Charles Gairdner Hospital Nedlands, Perth WA Australia

**Keywords:** apoptosis, immune checkpoint blockade, immunogenic cell death, radiotherapy

## Abstract

Recent clinical breakthroughs in cancer immunotherapy, especially with immune checkpoint blockade, offer great hope for cancer sufferers – and have greatly changed the landscape of cancer treatment. However, whilst many patients achieve clinical responses, others experience minimal benefit or do not respond to immune checkpoint blockade at all. Researchers are therefore exploring multimodal approaches by combining immune checkpoint blockade with conventional cancer therapies to enhance the efficacy of treatment. A growing body of evidence from both preclinical studies and clinical observations indicates that radiotherapy could be a powerful driver to augment the efficacy of immune checkpoint blockade, because of its ability to activate the antitumor immune response and potentially overcome resistance. In this review, we describe how radiotherapy induces DNA damage and apoptosis, generates immunogenic cell death and alters the characteristics of key immune cells in the tumor microenvironment. We also discuss recent preclinical work and clinical trials combining radiotherapy and immune checkpoint blockade in thoracic and other cancers. Finally, we discuss the scheduling of immune checkpoint blockade and radiotherapy, biomarkers predicting responses to combination therapy, and how these novel data may be translated into the clinic.

## Graphical abstract

Whilst the immunotherapy revolution has improved treatment prospects a growing number of cancer types, many people still receive no benefit. Growing evidence suggests that radiotherapy can improve the efficacy of immunotherapy. In this review, we discuss the mechanisms whereby radiotherapy can exert effects on the tumour immune microenvironment. We also discuss which radiotherapy strategies, both in the clinical and preclinical settings, have indicated additive or synergistic effects when combined with immunotherapy, in relation to dose, fractionation and relative timing.

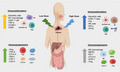

## Introduction

Immunotherapy, particularly immune checkpoint blockade (ICPB), is now used to treat a growing number of cancer types. Monoclonal antibodies targeting immune inhibitory receptors including anti‐programmed death receptor‐1 (anti‐PD‐1) and anti‐cytotoxic T‐lymphocyte‐associated antigen‐4 (anti‐CTLA‐4) can reverse T‐cell dysfunction and thus enhance tumor‐specific T‐lymphocyte activity.^1^ The introduction of ICPB into the clinic has yielded unprecedented results, with some patients experiencing dramatic tumor regression and long‐term survival, particularly in melanoma.^2^ However, resistance, relapse and tumor progression still eventually occur in many patients treated with ICPB. One potential determinant of primary resistance is whether or not the tumor microenvironment (TME) demonstrates an inflammatory infiltrate. Some tumors have an immunologically ‘cold’ microenvironment, which suggests that a pre‐existing antitumor immune response is lacking – making it difficult to treat, as response to ICPB appears to be more robust in the context of prior immunogenicity. On the one hand, a cold tumor is characterised by low or absent levels of tumor‐specific antigen, defective recruitment of antigen‐presenting cells (APCs), lack of co‐stimulation, and few infiltrating cytotoxic T cells.^3^ ‘Hot’ tumors, on the other hand, are characterised by high concentrations of tumor‐infiltrating immune cells, particularly CD3^+^CD8^+^ T cells at the tumor centre and invasive margin.^4^, ^5^ Accumulating data from preclinical studies suggests that radiotherapy can convert ‘cold’ tumors to ‘hot’ tumors – particularly through enhancing antigen visibility to dendritic cells (DCs). This 'licenses' DCs to present tumor antigens to naïve CD4^+^ T cells, or cross‐present to naïve CD8^+^ T cells, triggering cytokine and chemokine release plus activation and infiltration of tumor‐specific T cells to the tumor bed to target cancer cells.^6^ However, the successful combination of ICPB with radiotherapy requires exploration of optimal doses, fractionation, sequence, schedules and interval between ICPB and radiotherapy to maximise the synergistic effects of this approach. In this review, we briefly summarise the mechanism by which radiotherapy induces DNA damage, generates immunogenic cell death (ICD) and alters the characteristics of key immune cells in the tumor immune microenvironment, followed by an overview of recent preclinical and clinical data combining radiotherapy with ICPB.

## Induction of DNA damage and apoptosis after radiotherapy

Radiotherapy first entered the clinic in the early 1930s^7^ and remains one of the most widely used cancer treatment modalities. Many cancer patients will receive radiotherapy at some point during the course of their illness.^8^ Radiotherapy is usually delivered as X‐rays in the laboratory and outpatient clinic setting. These X‐rays are packets of energy travelling at the speed of light called photons, and they are generated in a linear accelerator. If the same high‐energy photons come from a natural source such as iridium 192, then the same particles are called γ‐rays. Although much less commonly used in practice, other larger particles that can be used to deliver radiation include protons and electrons, which can also be generated by a linear accelerator but have a different depth of penetration into tissue because of their bulkier mass than a photon. Ultimately, photons, protons, electrons and γ‐radiation have only slightly different cell kill effects on normal tissue and cancer for the same amount of energy deposited per unit mass. The SI unit of radiation is Gray (Gy), defined as the absorption of one joule of radiation energy per kilogram of matter. In the early days of its discovery, radiotherapy was delivered in single doses, which resulted in unacceptable toxicity to surrounding tissue. In 1911, Claudius Regaud proved that a ram's testicles could be sterilised without burning the scrotal skin by delivering a large dose broken into fractions over several days or weeks, thus inventing the concept of fractionated radiotherapy.^9^ Currently, doses of 1.8–2 Gy per fraction are considered standard fractionation, and that of > 2 Gy per days are considered hypofractionation. When the treatment is ‘delivered’ conformally to the target, either with additional equipment to immobilise the patient (e.g. using a stereotactic frame if treating the brain)or with imaging techniques to track the target during treatment (e.g. using a specialised robotic linear accelerator available commercially called the CyberKnife), then the treatment is called stereotactic radiotherapy (SRT). SRT results in less normal tissue receiving radiotherapy, so then the need for fractionation is reduced, depending on the proximity of the normal tissue to the stereotactic treatment. Using Regaud's example, SRT to the ram's testicles can be delivered in one fraction with immobilisation of the target, imaging and tracking during treatment to confirm its location before treating and using a plan that avoids the skin, thus negating the need for fractionated radiotherapy whilst achieving minimal skin toxicity. As SRT is now commonly available in the clinic, more and more patients with metastatic cancer have been treated with large fraction doses, and clinicians started to notice responses in other parts of the body, away from the site being treated presumably because of a systemic immunogenic effect of radiotherapy.

**Figure 1:**
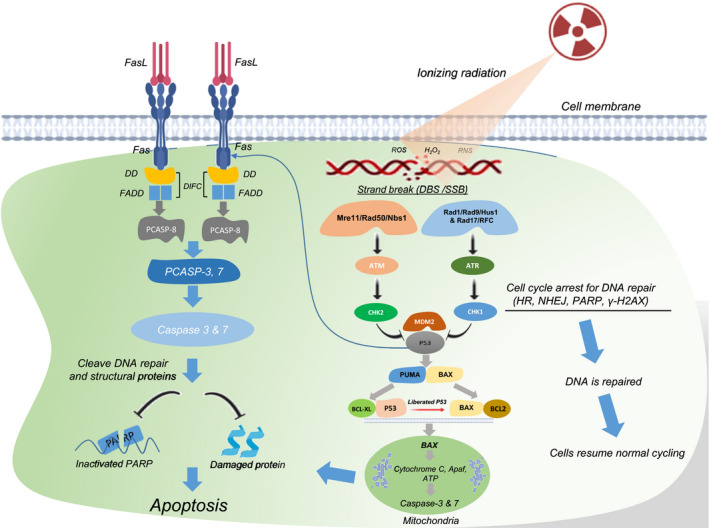
DNA damage and the programmed cell death pathway (apoptosis) induced by radiotherapy. Double‐ and single‐strand breaks induced by radiotherapy are sensed by Mre11/Rad50/Nbs1 and Rad1/Rad9/Hus1 and Rad17/RFC complexes, respectively. Mre11/Rad50/Nbs1 then recruits ataxia telangiectasia‐mutated protein (ATM), which phosphorylates checkpoint protein kinase 2 (CHK2). Rad1/Rad9/Hus1 and Rad17/RFC recruit ataxia telangiectasia and rad 3‐related (ATR), which phosphorylates checkpoint protein kinase 1 (CHK1). Activated CHK2 and CHK1 arrest cell cycle progression for DNA repair through homologous recombination (HR), nonhomologous end‐joining (NHEJ), poly (ADP‐ribose) polymerase (PARP) and γ‐H2AX. If DNA is repaired, cells will resume their normal cycles. However, if the damage is substantial, CHK2/CHK1 will phosphorylate P53 by dissociating P53 from mouse double minute 2 (MDM2), leading to the accumulation of transcriptionally active P53. The P53 then triggers the expression of pro‐apoptotic genes, namely p53‐upregulated modulator of apoptosis (PUMA) and BCL2‐associated X protein (BAX). PUMA disassembles complex P53 and anti‐apoptotic protein (BCL‐XL) in the cytoplasm. Liberated P53 disrupts pro‐apoptotic BAX and anti‐apoptotic BCL2 complex. Released BAX permeabilises mitochondrial outer membrane releasing cytochrome C, which binds to apoptotic protease‐activating factor (Apaf) and adenosine triphosphate (ATP) to form apoptosome and activate caspase‐9 and finally effector caspase‐3 and caspase‐7 inducing intrinsic apoptosis. Moreover, P53 also transactivates the death receptor (Fas/CD95) and death ligand (FasL/CD178). The interaction of Fas and FasL leads to trimerisation of CD95 and clustering of intracellular death domain (DD). DD then recruits FAS cell‐surface death receptor‐associated death domain (FADD). The FADD activates procaspase‐8 (PCASP‐8) forming death‐inducing signalling complex (DISC), which will further produce effector caspase‐3 and caspase‐7 cleaving DNA repair proteins such as PARP, structural protein, inducing membrane blebbing and DNA fragmentation leading to extrinsic cell death.

Radiotherapy directly induces abasic lesions, deoxyribose ring openings, single‐ and double‐strand DNA breaks, RNA, lipid and protein cross‐linking. Moreover, it indirectly damages DNA by generating reactive oxygen and nitrogen species.^10^, ^11^ DNA damage induced by radiotherapy activates a series of events collectively named DNA damage responses, including the recognition of DNA damage, activation of checkpoints and cell cycle arrest,^12^ to restore genomic integrity (Figure 1). Durante and Formenti^13^ suggest that one possible underlying mechanism may be through micronuclei expression, which influences the Trex1‐STING pathway when larger doses per fraction are administered, generally more than 8 Gy per fraction.

## Generation of immunogenic cell death after radiotherapy

Tumor cell death, through apoptosis and other cell death pathways induced by radiotherapy, generates cellular debris and release of damage‐associated molecular patterns (DAMPs) and can occur in a manner that induces an adaptive antitumor immune response known as ICD. This may result in an improved immune response and lead to enhanced control of tumor growth, especially in the context of ICPB.

**Figure 2:**
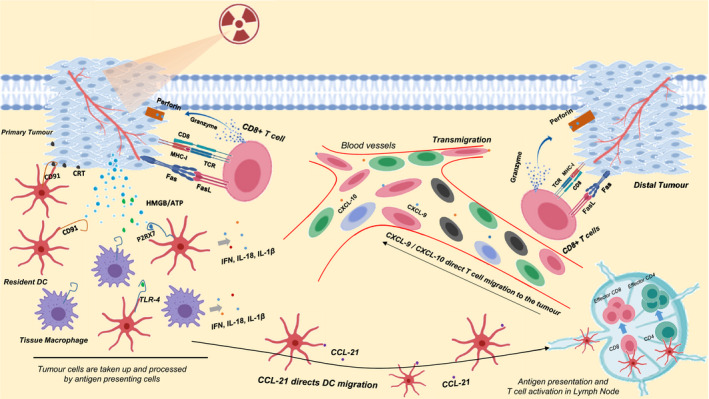
Induction of immunogenic cell death after radiotherapy. Tumor cells are treated with radiation. The dying tumor cells then induce the translocation of damage‐associated molecular patterns (DAMPs) such as CRT to plasma membrane of the cells and release other DAMPs such as high mobility group box‐1 protein (HMGB‐1) and adenosine triphosphate (ATP) into the immune milieu. Resident dendritic cells (rDCs) interact with CRT via CD91 receptor, leading to the phagocytosis of tumor cells to generate peptide antigen. rDCs migrate to draining lymph nodes under the direction of chemokine – CCL‐21 – to present tumor antigen to naïve CD4^+^ T cells and cross‐present the antigen to CD8^+^ T cells through major histocompatibility classes (MHC)‐I and MHC‐II, respectively. Effector T cells, particularly CD8^+^ T cells precisely directed by chemokines – CXCL‐9 and CXCL‐10 – home in on the tumor, killing it by inducing apoptosis through granzyme/ perforin pathway and Fas/ Fas‐ligand interaction. HMGB‐1 and ATP also bind to TLR‐4 and P2RX7, respectively, on dendritic cells/ tissue macrophages, inducing the activation of these cells to release pro‐inflammatory cytokines such as interferon, interleukin (IL)‐1β and IL‐18 to inflame the environment for more robust antitumor immune responses.

Three major DAMPs contribute to the generation of ICD. Firstly, calreticulin (CRT), an endoplasmic reticulum (ER) chaperone, is found on the outer leaflet of the dying tumor cells. Here, CRT acts as an ‘eat me’ signal to APCs, particularly DCs and macrophages, through CD91 ‐ leading to the release of pro‐inflammatory cytokines such as tumor necrosis factor α (TNF‐α) and interleukin‐6 (IL‐6).^14^, ^15^, ^16^ The CRT‐CD91 interaction also mediates APC recruitment to the tumor, followed by phagocytosis of tumor cells by DCs and efficient tumor antigen presentation to T cells, ultimately resulting in the activation of antitumor immune responses.^16^ Secondly, high motility group box‐1 (HMGB‐1), a pro‐inflammatory mediator, is released into the immune milieu from dying, necrotic, damaged tumor cells where it engages with Toll‐like receptor (TLR)‐4 on DC/tissue macrophages to promote the transcription of inflammatory genes, inducing inflammatory responses in the TME.^17^, ^18^ A third DAMP is ATP, which binds to purinergic P2RX7 receptors on DCs, leading to the activation of NLRP3/ASC/caspase‐1 inflammasome, and eventually induces the production of IL‐18 and IL‐1β. Secreted IL‐1β is required to prime interferon (IFN)‐γ‐producing tumor antigen‐specific CD8^+^ T cells, necessary for effective antitumor immunity and to initiate further pro‐inflammatory events^17^, ^19^ (Figure 2). Overall, the induction of ICD is an essential pathway to activate antitumor immunity in the setting of an otherwise poorly immunogenic tumor. Immunogenic DAMPs released as a consequence of radiotherapy act as potent inducers to prime innate and adaptive antitumor immune responses. However, the induction of ICD does not always abrogate tumor growth – and may even induce tumor progression in some cancers.^20^, ^21^, ^22^ Hence, determining the optimal doses, fractions and schedules of radiotherapy may help counter the negative effects of DAMPs after radiotherapy.

## Enhancing abscopal responses: Single or multisite radiotherapy?

The ICD generated by radiotherapy, in some cases, can activate systemic immune responses and result in control of non‐irradiated distant metastatic lesions away from a target that was irradiated – a phenomenon known as the abscopal response. The abscopal response was first reported in 1953 when Dr R.H mole found the regression of a tumor outside the irradiated site, following radiotherapy to a single site in an individual with metastatic disease.^23^ However, it is relatively rare in the clinic and it has been difficult to consistently reproduce abscopal effects in preclinical studies.^24^ Despite this rare occurrence, single lesion irradiation continues to be the cornerstone for radiotherapy and ICPB combination in most current clinical trials.^25^ However, it is also important to understand whether multiple lesion radiotherapy provides any benefit over single lesion radiotherapy in generating abscopal responses. Tumors are heterogeneous,^26^, ^27^, ^28^ suggesting that tumor‐associated antigens (TAAs) present in some tumor sites might be different from those in other tumor sites, or may not be equally immunogenic. Targeting a single metastatic site in patients with multiple metastases is unlikely to unmask TAA in another site unless those TAAs are shared, thus useful antitumor immune responses may not be activated systemically. Theelen *et al*.^29^ reported stereotactic body radiotherapy on a single tumor site before pembrolizumab enhanced overall response rates, but did not provide meaningful clinical benefit. Moreover, in a multicentre, randomised, double‐blinded, phase 3 trial using anti‐CTLA‐4 or placebo following radiotherapy to a single lesion, patient outcomes were no better in those receiving anti‐CTLA‐4 than placebo.^30^

The effects of radiotherapy on the TME, including remodelling vasculature^31^ and reducing suppressive immune cells such as myeloid‐derived suppressor cells,^32^ provide another rationale for multisite radiotherapy. Given that the TME in each anatomical location may be different, and that local effects of radiotherapy may be as or more important than enhancing the systemic immune response, multisite radiation may restore the TME and mediate T‐cell access to tumors. Furthermore, irradiating some anatomical sites may facilitate more immune activation than others. For example, in patients with non‐small cell lung cancer (NSCLC), irradiation of liver metastases induced greater activation of antitumor immunity than pulmonary metastasis irradiation.^33^ Several other case reports revealed multiple target radiation produced abscopal responses in breast cancer,^34^ renal pelvis urothelial carcinoma,^35^ metastatic cholangiocarcinoma,^35^ metastatic non‐small cell lung cancer^36^ and multiple metastatic lung adenocarcinoma.^37^ Taken together, multiple target irradiation tends to induce greater antitumor immunity and generate frequent abscopal responses than single target. Therefore, this approach should be investigated further in clinical settings.

## Effects of radiotherapy on the tumor immune microenvironment

**Figure 3:**
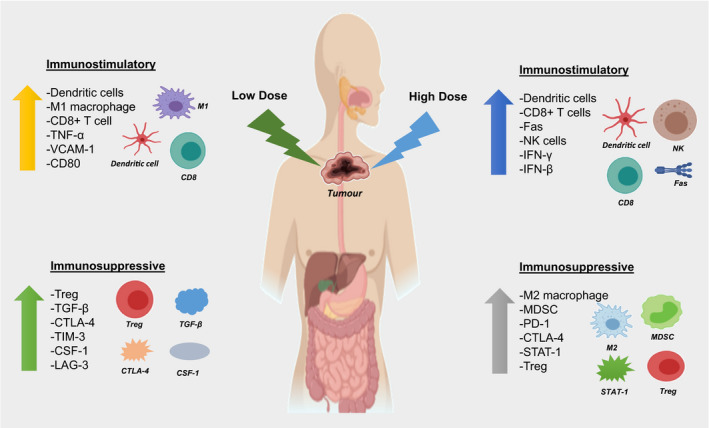
Effects of low‐ and high‐dose radiotherapy on tumor immune microenvironment. Both low‐ and high‐dose radiotherapies drive the upregulation of immunostimulatory (e.g. CD8^+^ T cells, NK cells) and immunosuppressive immune cells (e.g. Treg, MDSC, M2 macrophage) but influence differing cell subsets.

The ability to escape immune surveillance is a hallmark of cancer.^38^ Radiotherapy is capable of enhancing antitumor immune responses by converting poorly immunogenic tumors into more highly immunogenic ones, not only through ICD, but also through the modification of the characteristics of key immune cells within the TME (Figure 3). The next sections explore a number of specific effects of radiotherapy on the immune system in detail.

### Polarising macrophages and enhancing their function

Macrophages can contribute roughly 50% of the tumor mass, and their accumulation correlates with poor prognosis in most cancers.^39^ Tumor‐associated macrophages (TAMs) enhance tumor growth and promote angiogenesis and immunosuppression. TAMs may also be associated with recurrence after conventional cancer therapies (e.g. chemotherapy).^39^

Radiotherapy can alter the polarisation and function of macrophages in preclinical studies. In a murine model of spontaneous insulinoma, low‐dose irradiation (2 Gy) in late‐stage tumor Rip‐Tag5 mice induced M1 TAM‐associated cytokines (e.g. TNF‐α, IL‐12p70 and IFN‐γ), and suppressed p38 mitogen‐activated protein (p38MAPK), leading to the acquisition of M1 macrophage phenotype.^40^ Similarly, delivering lower radiation doses (0.5–2 Gy) to mice bearing human pancreatic carcinoma can programme macrophage differentiation into an inducible nitric oxide (NO) synthase (iNOS) M1 phenotype, mediating the recruitment of cytotoxic T‐lymphocytes (CTLs) to the tumor via iNOS by stimulating endothelial activation and Th1 chemokine expression and reducing the production of immunosuppressive, angiogenic and tumor growth factors.^41^ Moreover, low‐dose total body irradiation (2 Gy) of Rip‐Tag (RT5) transgenic mice bearing spontaneous insulinoma decreases hypoxia‐inducible factor 1 (HIF‐1) and switches irradiated TAMs towards an M1 phenotype.^42^

High‐dose radiotherapy, however, promotes M2 macrophage polarisation in many tumor models. Irradiation (12 Gy) of oral squamous cell carcinoma‐bearing mice induced intratumoral recruitment of CD11b^+^ myeloid cells, which were differentiated into an M2‐like phenotype under a hypoxic microenvironment. This may contribute to tumor progression and recurrence as M2 macrophages can promote tumor vasculogenesis.^43^ Additionally, whole‐brain irradiation of ALTS1C1 glioblastoma‐bearing mice (8 or 15 Gy) led to tumor regression, but increased monocyte‐derived TAMs.^44^ In a pancreatic ductal adenocarcinoma model, mice treated with 2–12 Gy exhibited marked increases in TAMs with an M2 phenotype – as evidenced by lower MHC‐II expression and increased CD204 and CD206 expression – leading to T‐cell suppression.^45^ Overall, preclinical data indicate that low radiation doses may modulate macrophages towards a pro‐inflammatory phenotype (M1), essential for antitumor immune responses, whilst high radiotherapy doses may favor the polarisation of macrophage towards M2 and thus may contribute towards a more tumor‐permissive microenvironment.

### Enhancing dendritic cell maturation and antigen presentation

Dendritic cells are professional APCs, capable of instigating immune responses and bridging innate and acquired immunity.^46^ DCs are critical to generating tumor‐specific T cells, thereby improving antitumor immune responses and inhibiting tumor growth. Many studies have shown that DC function is impaired in cancer, inhibiting the activation of tumor‐specific T cells.^47^, ^48^

An emerging body of evidence suggests that radiotherapy can restore DC function. In a mouse model of B16 melanoma in C57BL/6 mice, a single dose of 10 Gy‐induced expression of co‐stimulatory receptors (CD70 and CD86) enhanced T‐cell priming on live CD45^+^CD11c^high^MHC‐II^high^ cells and markedly increased leucocyte accumulation 7 days after radiation.^49^ Similarly, increased peptide‐presenting CD11c^+^ DC in draining lymph nodes (DLNs) 5 days after receiving 20 Gy was observed in lung cancer (A549)‐bearing mice, with increased intratumoral CD11c^+^TCR tetramer^+^ cells having an enhanced ability to stimulate T cells. There was also a significant increase in MHC‐II molecules on mDCs within 48 hours post‐irradiation, suggesting radiotherapy may have activated DCs at the tumor site and promoted their maturation and migration to DLN to prime naïve T cells.^50^ In an *ex vivo* investigation, purified splenic DCs from irradiated C57Bl/6 mice (0.25 Gy) cultured with ovalbumin (OVA) protein had a 1.5‐fold increase in OVA peptide uptake compared to a lower radiation dose of 0.1 Gy. In this study, the treatment of purified DCs with 0.1 Gy mildly increased IL‐1β, IL‐6 and IL‐10 gene expression, whilst 0.2 and 0.25 Gy upregulated gene expression of all studied cytokines in splenic DCs, including IL‐1β, IL‐6, IL‐10, IL‐12 and TNF‐α. Interestingly, irradiated purified DCs also inhibited regulatory T‐cell (Treg) proliferation, which may enhance effector T‐cell activation/proliferation.^51^ In a dual tumor model, low‐dose total body irradiation (0.1 Gy) combined with hypofractionated irradiation (8 Gy × 3) of BALB/C‐derived mammary carcinoma 4T1 cells increased the number of CD86^+^ DC cells in the secondary tumor.^52^ DC expression of CD86 is a critical step in T‐cell activation, as CD86 expressed on DCs will ligate with C28 on naïve T cells, providing essential co‐stimulatory signals. In another study, inoculation of mice with Lewis lung cancer cells irradiated to 8 Gy (IR‐LLC) promoted DC maturation and increased the proportion of CD4^+^ T cells in the spleen.^53^ In summary, extensive preclinical data indicate that radiation induced inflammatory responses enhancing DC infiltration and function, and thus promote the activation of antitumor immunity.

### Promoting and inhibiting myeloid‐derived suppressor cells

Myeloid‐derived suppressor cells (MDSCs) exert suppressive functions through either production of NO from iNOS or increased arginase‐1 expression, resulting in T‐cell cell cycle arrest and inactivation.^54^

Intratumoral MDSCs have been observed in many cancers and may confer resistance to immunotherapy^54^, ^55^; there is evidence from both murine and human studies that radiotherapy may also affect MDSC numbers and function. In a tumor model of M38 colon cancer, up to threefold increase in the level of monocytic Ly6C^hi^ myeloid cells (CD11b^+^) among total CD45^+^ cells was found in irradiated tumor (20 Gy) compared to shame irradiation control 3 days after radiotherapy, suggesting Ly6C^hi^ myeloid cells may alter the inflammatory profile in the TME and therefore may reduce the antitumor effects of radiotherapy.^56^ When radiotherapy and chemotherapy were combined in patients with stage III–stage IV head and neck squamous cell carcinoma (HNSCC), there was a significant increase in polymorphonuclear MDSC population from PBMC at weeks 2 and 7 of treatment, with detectable STAT‐3 and PD‐L1 expression. This was coupled with a transient increase in the plasma level of arginase – an immunosuppressive enzyme produced by MDSC, inhibiting T‐cell activities. An increase in chemokine receptors (CCL2/MCP1) critical for the recruitment of MDSCs was also reported after 7 weeks of this combined modality therapy.^57^ Therefore, the effects of any potential immunostimulation from radiotherapy in HNSCC may concurrently be reduced by STAT‐3 signalling pathway, PD‐L1 upregulation and CCL2/MCP1 expression on MDSC. This raised the possibility that targeting STAT‐3, PD‐L1 and CCL2/MCP1 may enhance responses to radiotherapy.

Radiotherapy has also been reported to reduce MDSC numbers, generally at higher doses rather than fractionated lower doses.^58^ This may in turn benefit the T‐cell milieu. A study from Filatenkov and colleagues^32^ revealed that higher single fractions (30 Gy) reduced the proportion of intratumoral MDSCs, with a subsequent intense CD8^+^ T‐cell infiltration in CT26 and MC38 colon cancer cell lines. These data support the fact that the effects of radiation promoting or inhibiting MDSCs depend on the radiotherapy dose fraction size.

### Increased activation and infiltration of tumor‐specific CD8^+^ T cells

CD8^+^ T cells function primarily to screen peptide antigen presented by MHC class I molecules.^59^ CD8^+^ T cells kill infected cells and tumor cells by inducing apoptosis through Fas/FasL interaction and the perforin and granzyme B pathways.^60^, ^61^ Many studies report radiotherapy enhanced the activation and tumor infiltration of CD8^+^ T cells.^62^ For example, in irradiated C57BL/6 mice bearing B16gp melanoma tumors, a single dose of 10 Gy led to a substantial increase in the percentage of infiltrating CD45^+^ T cells and tumor‐specific CD8^+^ T cells 7 days after irradiation compared to untreated tumors. When CD8^+^ T cells were depleted from mice bearing B16gp tumors 1 day before radiotherapy, the therapeutic effects of radiotherapy were completely abolished, suggesting the presence of CD8^+^ T cells during or immediately after radiotherapy was critical for treatment efficacy in this model.^49^ In a model of colon cancer, a huge influx of tumor‐specific CD8^+^ T cells (70% in irradiated tumor vs. 19% in non‐irradiated tumor) and loss of MDSC were observed after high‐dose single fraction of 30 Gy – an effect which was dependent upon DC cross‐presentation, secretion of IFN‐γ and CD40 ligand (CD40L)‐expressing CD4^+^ T cells. However, fractionated radiotherapy (10 daily doses of 3 Gy) did not induce robust CD8^+^ T‐cell infiltration with only 10% displaying tumor control at day 100, illustrating that fractionated radiotherapy did not improve the therapeutic effects compared to higher‐dose radiotherapy in this model.^32^ Moreover, the treatment of C57BL/6 mice bearing dual B16 (parental tumor) and B16 radiotherapy‐resistant melanoma tumor with 30 Gy in two fractions to the resistant lesion resulted in a substantial increase in CD8^+^ T cells with effector phenotype (CD44^+^CD62L^−^) in the untreated parental tumor; however, the proportion of CD8^+^ T cells remained lower in the treatment‐resistant tumor.^63^ A prospective study examining resected tumors from early‐stage lung cancer patients reported changes in both CD4^+^ and CD8^+^ T cells after stereotactic ablative radiotherapy (SABR), with the detectable expression of GATA‐3, T‐bet and ROR‐γt – transcription factors of Th2, Th1 and Th17 CD4^+^ T cells, respectively, and a reduction in CD4^+^ Foxp3^+^ regulatory T cells (Tregs). This suggests SABR augmented systemic antitumor immune responses by increasing the number of pro‐inflammatory/killer cells – particularly CD4^+^ and CD8^+^ T cells.^64^ In addition, combining low‐dose total body irradiation (L‐TBI) of 0.1 Gy at a dose rate of 24 cGy min^−1^ with hypofractionated therapy (HFT) of 8 Gy × 3 at a dose rate of 400 cGy min^−1^ resulted in greater levels of activated CD8^+^ T cells in secondary tumors, along with high CD8^+^ IFN‐γ^+^ T cells and reduced granulocytic MDSC and M2 macrophages. To confirm that CD8^+^ IFN‐γ^+^ T cells induced by combined L‐TBI and HFT contributed to the suppression of tumor growth and metastasis, CD8^+^ T cells were depleted – whereupon reduced suppressive effects of the combination therapy were observed, suggesting the responses were dependent on CD8^+^ T cells.^52^ In NSCLC, six patients treated with hypofractionated SRT (48 Gy in six or eight fractions) had substantial increases in CD8^+^ T cells 3 weeks post‐SRT. Those CD8^+^ T cells were activated, with higher TNF‐α^+^ CD8^+^ T cells, IFN‐γ^+^ CD8^+^ T cells and IL‐2^+^CD8^+^ T cells along with reduced frequencies of Tregs after SRT, suggesting hypofractionated SRT increased cytotoxic activities of immune cells, and down‐modulated immune suppressive cells, leading to reduced tumor growth.^65^ The acknowledged critical role of CD8^+^ T cells in the systemic and local antitumor response highlights the importance of understanding how radiotherapy specifically affects this lymphocyte subset.

### Increasing natural killer cytotoxicity

Natural killer (NK) cells are critical in the first line of defence against tumor and viral infection.^66^ NK cells can recognise distressed cells or cells with reduced surface MHC expression; they are also major killers of pathogen‐infected or tumor cells.^67^

Radiotherapy has been shown to enhance NK cell function and cytotoxicity. In preclinical studies, peripheral blood NK cells (CD5^dim^, NKp46^+^) co‐cultured with irradiated K562‐C9‐mIL21 feeder cells underwent a massive expansion with over 90% of expanded NK cells expressing granzyme B and IFN‐γ. Further analysis at days 14 and 21 revealed that the expanded NK cells had enhanced capacity to lyse tumor cells in a dose‐dependent manner, leading to delayed tumor growth.^68^ *In vivo* experiments immunising C57BL/6 mice with irradiated B16‐F‐10 melanoma (15 Gy) showed a significant accumulation of CD3^−^NK1.1^+^ NK cells with a substantial increase in the immune regulatory NK cell subpopulation CD27^+^CD11b^−^.^69^ Another study led by Lee *et al*.^70^ examining cytotoxic NK cell expansion demonstrated that 25 Gy radiation induced upregulation of co‐stimulatory receptors (NKG2D) critical for NK cell activation. In this study, low‐dose radiotherapy of 75 mGy (12.5 mGy min^−1^) increased NK cell infiltration and induced strong cytotoxicity compared to sham irradiation. In a murine model of sarcoma, BALB/c mice irradiated with 0.1 and 0.2 Gy (single dose) suppressed metastasis, mainly because of the stimulation of NK cell‐mediated cytotoxic activity.^71^

### Increased regulatory T cells with a highly suppressive function

CD4^+^ Tregs function primarily to suppress immune responses and thus maintain homeostasis and tolerance.^72^ High tumor Treg infiltration has been reported in ovarian, liver, melanoma and oesophageal cancers, and is associated with an aggressive phenotype (extensive review by Jørgensen *et al*.^73^).

Many studies have shown that radiotherapy can increase the presence of Tregs in the TME.^65^, ^74^ In a preclinical study of heterotopic prostate cancer, local radiation with single or fractionated dose of 2 Gy led to increased Tregs in the spleen 2 days later, which was dose‐dependent. Increasing the dose to 20 Gy doubled the proportion of tumor‐infiltrating CD4^+^ Tregs, suggesting Tregs were more radioresistant than other cells, and may mediate immune evasion during treatment.^75^ In a model of head and neck cancer, *ex vivo* irradiation (10 Gy) of CD4^+^ T cells isolated from mouse spleen induced pSTAT‐3 expression correlated with increased Tregs and TGF‐β, suggesting radiotherapy mediated the conversion of CD4^+^ T cell to Tregs. This may contribute to radioresistance, and limit therapeutic outcomes, since Tregs can inhibit DC maturation and T‐cell activation, potentially leading to impaired antitumor immune responses.^76^ Additionally, the treatment of mice in various models of melanoma, kidney and colorectal cancer using stereotactic irradiation (10 Gy) increased frequencies of Tregs in all models, with highly suppressive phenotypes characterised by higher expression of helios, CTLA‐4 and 4‐1BB expression in irradiated mice than non‐irradiated mice. In this study, Tregs proliferated more robustly than other T cells in the TME, suggesting radiotherapy promoted Treg proliferation; hence, using Treg‐targeting agents along with radiotherapy may limit the immunosuppressive activities of Tregs and improve responses to radiotherapy.^77^ Besides, the frequencies of Tregs – plus the ratio of Tregs to CD8^+^ T cells – were increased in the primary and secondary tumors 7 days after radiotherapy; this phenomenon was regulated by CTLA‐4 blockade in a mesothelioma model.^78^ Taken together, in mouse models radiotherapy often leads to Treg induction, which may hamper antitumor immunity. Hence, inhibiting the negative effects of Tregs may restore the efficacy of radiotherapy.

## Increased cytokines, adhesion proteins and upregulated inhibitory molecules

In addition to changing the characteristics of immune cells in the TME, radiotherapy also impacts the production of cytokines and upregulates the expression of adhesion molecules plus inhibitory receptors/ligands, which can be either immunostimulatory or immunosuppressive. A study by Gerber and colleagues^79^ demonstrated that 7 Gy local irradiation of mice bearing colon cancer resulted in a greater increase in IFN‐γ production on day 9, which remained elevated until day 11 post‐radiotherapy. The level of IFN‐γ also correlated with tumor regression and lytic capabilities of CD8^+^ T cells. However, CD8^+^ T‐cell depletion reduced IFN‐γ levels by more than 90% and completely abolished radiation effects. This suggests that IFN‐γ production is crucial in mediating antitumor effects by CD8^+^ T cells after radiotherapy. In another study, using B16 tumor‐bearing mice, radiotherapy treatment upregulated IFN‐β at RNA and protein levels; further analysis using cell sorting revealed tumor‐infiltrating CD45^+^ cells were the main producers of IFN‐β in irradiated mice. In this study, IFN‐β induced massive expansion of antigen‐specific T cells with increased lytic capabilities critical to control tumor growth.^80^

Increased endothelial and tumor cell expression of adhesion proteins including intracellular adhesion molecules (e.g. I‐CAM, E‐selectin), CD44, integrin α4, integrin α5, and integrin β1 has been reported after radiotherapy treatment with doses between 2 and 20 Gy.^81^, ^82^, ^83^, ^84^, ^85^ Moreover, increased expression of inhibitory ligands, particularly PD‐L1, has also been shown in various cancers with doses ranging from 5, 10 and 50 Gy.^86^, ^87^, ^88^ Additionally, inhibitory receptors such as PD‐1, CTLA‐4, glucocorticoid‐induced TNFR family‐related gene (GITR), T‐cell immunoglobulin and mucin domain‐containing‐3 (TIM‐3), and lymphocyte‐activation gene 3 (LAG‐3) were also reported to upregulate on CD4^+^ and CD8^+^ T cells in many animal and human cancer studies with radiotherapy doses ranging from 1.8 to 30 Gy.^32^, ^33^, ^77^, ^89^, ^90^ This may drive an inhibitory/suppressive tumor environment and hinder T‐cell function, presenting a major obstacle for effective therapy. However, combining radiotherapy with ICPB may overcome this suppressive effect and allow T cells to work more effectively during radiotherapy.

## Normalisation of tumor vasculature and reduction in tumor hypoxia

Tumor growth and progression are supported by the generation of new tumor blood vessels. Tumor blood vessels are heterogeneous and morphologically abnormal in their structure, organisation and function and can be distinguished from normal vasculature through numerous cell‐surface molecules and extracellular matrix proteins, which are mostly associated with stimulating tumor angiogenesis; this may confer treatment resistance.^91^, ^92^ Low‐dose fractionated radiotherapy (2 Gy, five fractions per week) has been reported to normalise tumor vasculature,^31^ which may enhance perfusion and intratumoral drug delivery.

In addition, the chaotic, morphologically and functionally deformed microvasculature reduces oxygen delivery to tumor cells, whilst available oxygen is utilised by rapidly growing tumor cells. This leads to decreased oxygenation (hypoxia) in the TME.^93^, ^94^ Hypoxia can promote tumor progression and metastasis via direct and indirect mechanisms. Ten randomly selected HNSCC patients from 102 patients enrolled in a FMISO‐PET imaging research study and, with varying hypoxic tumor scores, were treated with an additional radiation boost dose of 10 Gy, higher doses demonstrating a significant improvement in tumor control probability.^95^ A systematic review and meta‐analysis in HNSCC revealed that hypofractionated radiotherapy led to more pronounced modification of tumor hypoxia, but was associated with more complications.^96^ A study using a nanotheranostic system to enhance the effects of X‐ray radiation revealed that X‐rays promoted high level of reactive oxygen species and NO, improved hypoxia through NO‐induced vasodilation and reduced HIF‐1α; they also induced DNA damage and cell death on hypoxic cells, leading to better tumor control.^97^ Conventional radiotherapy (1.2–3 Gy per fraction) could reoxygenate the hypoxic cells and thus is considered effective to kill hypoxic tumor cells.^98^ Hence, these data indicate that radiotherapy can reduce tumor hypoxia, which may inhibit tumor cell proliferation, and metastasis.

## Tissue‐resident memory cells in the context of immune checkpoint blockade and radiotherapy

Tissue‐resident memory cells (T_RM_) are non‐recirculating immune cells, which reside in peripheral tissues to protect the host against infection and cancer.^99^, ^100^ Although CD4^+^ T_RM_ cells have been identified in both healthy tissue^101^ and tumors,^102^ their functions are less well‐characterised and only CD8^+^ T_RM_ cells are associated with improved outcomes in cancer. CD8^+^ T_RM_ cells are identified by the co‐expression of CD69^+^CD103^+^,^99^, ^100^ and they may prevent the development of clinically relevant cancer through tumor immune equilibrium and CD103‐enhanced tumor cell killing.^99^

Tumor‐specific T_RM_ cells express a wide range of immune checkpoint molecules including PD‐1, TIM‐3 and LAG‐3,^103^, ^104^, ^105^ suggesting T_RM_ may serve as an important target for ICPB therapy. Analysis of tumor biopsies from advanced‐stage metastatic melanoma patients treated with anti‐PD‐1 monotherapy revealed the expansion of T_RM_ early during treatment for the majority of patients, with a trend towards greater expansion in responders than non‐responders to PD‐1 blockade.^106^ Interestingly, *ex vivo* PD‐1 blockade on T_RM_ isolated from patients with lung carcinomas greatly promoted lytic activity against tumor cells.^107^ However, ICPB treatment may also be associated with T_RM_‐related hyper‐inflammation. A recent study in a mouse model revealed that PD‐1 and TIM‐3 blockade worsened skin inflammation caused by T_RM_.^108^

The interaction of T_RM_ with radiotherapy alone is less well‐understood, and most studies combined radiotherapy with other therapies. In a preclinical study of cervical cancer, the combination of a cancer vaccine (HPV E6/E7) with local X‐ray radiation increased the number of intratumoral CD103^+^CD8^+^ T cells and was also associated with increased treatment efficacy.^109^ Moreover, combining fractionated radiotherapy and anti‐PD‐1 treatment enhanced antitumor immunity, which was mediated by T_RM_ and infiltrating T cells.^110^ In both preclinical and clinical studies, the presence of T_RM_ is associated with improved outcomes and may be a potential biomarker of response to combination radio‐immunotherapy. However, more studies are warranted to validate this hypothesis.

## Combining radiotherapy with immune checkpoint blockade

As ICPB does not result in tumor regression in more than half of patients for the majority of solid tumor types, finding strategies to enhance the likelihood of response is critically important. Many studies are therefore investigating multimodal approaches by combining ICPB with conventional cancer therapies to increase response rates. Radiotherapy is considered a key candidate to partner with ICPB, because of its ability to release DAMPs, activate immune responses, potentially turning ‘cold’ tumors into ‘hot’ tumors. Being a local treatment, radiotherapy also allows the avoidance of many unwanted systemic side effects and toxicities. Until recently, studying focal radiotherapy and checkpoint blockade was challenging as animal studies were performed in off‐duty human‐use linear accelerators. However, dedicated small animal irradiation facilities are becoming increasingly available and have allowed for rapid expansion of this work.

## Preclinical studies combining radiotherapy and checkpoint blockade

### Thoracic cancers

In view of the potential immunogenic effects of ICPB, one key question is whether focal radiotherapy enhances the efficacy of this systemic treatment. A number of investigators have studied this in thoracic cancer models, although varied approaches have been used. In a C57BL/6 tumor xenograft mouse model of lung cancer, the addition of local 6 Gy in three daily fractions to anti‐PD‐L1 treatment effectively inhibited tumor progression compared to either anti‐PD‐L1 or radiotherapy alone, suggesting synergy between radiotherapy and anti‐PD‐L1. Increased intratumoral CD8^+^ T cells and reduced MDSC and iTregs were also found after this combinatorial approach, as compared with either single‐agent therapy.^111^ In another NSCLC mouse model, the combination of precise target image‐guided radiotherapy 8.5 Gy in two fractions with anti‐PD‐1 showed a significant (70%) reduction in tumor volume compared with baseline, and durable tumor regression for up to 12 weeks.^112^ In a dual murine model of mesothelioma, left (primary tumor) and right flanks (secondary tumor), combining 5 Gy local γ‐irradiation with anti‐CTLA‐4 antibody, increased antitumor effects over either single agent. This study also showed that radiotherapy alone increased both Treg and cytotoxic T‐cell infiltration into primary and secondary tumors. However, the proportion of Tregs to effector T cells was reversed by the addition of anti‐CTLA‐4 with increased CD8^+^ T‐cell activation.^78^ Notably, each of these studies used different doses, schedules and methodologies, with no detailed optimisation of the radiotherapy component of the investigation.

### Other cancers

Additive or synergistic effects between radiotherapy and ICPB have been reported in several other preclinical studies (Table 1). In a model of osteosarcoma, a single 5.3 Gy fraction carbon ion beam irradiation to the leg on one side only stopped tumor growth by day 21, although tumors started to regrow on day 24. However, the addition of dual checkpoint blockade (anti‐PD‐1 and anti‐CTLA‐4) to radiotherapy on days 9, 12 and 15 prolonged antitumor effects. Combination therapy promoted greater than twofold radiosensitivity on day 33, indicating immune checkpoint inhibitors were radiosensitising. In this study, increased CD8^+^ granzyme B^+^ T cells and CD4^+^ T cells in non‐irradiated areas were also found after combination treatment.^113^ Additionally, in glioma‐bearing mice treated with combinations of anti‐PD‐1, anti‐TIM‐3 or local 10 Gy stereotactic radiation, dual therapies increased median survival modestly over single agents. Interestingly, triple‐therapy anti‐TIM‐3/anti‐PD‐1/SRS resulted in overall survival of 100%. Furthermore, intratumoral infiltration with IFN‐γ^+^CD4^+^ and IFN‐γ^+^CD8^+^ T cells was significantly higher in combination therapy groups as compared with any single agents.^114^ In a mouse model of Lewis lung carcinoma, adding anti‐CTLA‐4 antibody increased the antitumor efficacy of (30 Gy) single‐fraction local X‐ray irradiation (13–19 days) compared to radiation alone and survival time was also longer in dual‐therapy group compared to radiation alone.^115^ These promising preclinical studies strongly suggest synergy or at least additive efficacy between radiotherapy and ICPB, encouraging translation into clinical studies.

**Table 1:** Selected preclinical studies combining radiotherapy with ICPB

Type of cancer	Radiotherapy dose	ICPB agent	Outcomes	Study
Melanoma	8 Gy (4 fractions)	Anti‐PD‐1	Reduced tumor growth; large number of activated CD8^+^ T cells even in non‐irradiated areas suggesting abscopal effects	Pfannenstiel *et al*.^136^
Head and neck squamous cell carcinoma	10 Gy	Anti‐PD‐L1	Enhanced tumor growth control and improved survival compared to either radiotherapy or anti‐PD‐L1 alone	Oweida *et al*.^137^
Hepatocellular carcinoma	10 Gy (single fraction)	Anti‐PD‐L1	Greatly suppressed tumor growth compared with PD‐L1 or radiotherapy alone; increased CD8^+^ T cells and restored its function	Kim *et al*.^138^
Pancreas tumor	12 Gy (3 fractions, daily)	Anti‐PD‐L1	Delivered radiotherapy booster shot after ICPB to second tumor significantly reduced tumor growth at third non‐irradiated area. This was associated with transient increase in CD4^+^, CD8^+^ T cells, MDSC and TAM	Chuong *et al*.^139^
Glioma	10 Gy (single fraction)	Anti‐CTLA‐4	Induced at least 50% long‐term tumor‐free survival with high CD4^+^ and CD8^+^ tumor‐infiltrating T cells	Belcaid *et al*.^140^
Colon cancer	10 Gy (single fraction)	Anti‐CTLA‐4 Immature DC	Inhibition of distant tumor by IR/iDC and this effect was enhanced by the addition of anti‐CTLA‐4; survival rate has also been improved with tumor‐specific interferon‐γ‐producing T cells and cytotoxic T‐cell activity	Son *et al*.^141^
Non‐small cell lung cancer	24 Gy (3 fractions)	Anti‐PD‐1	Increased inflammation in treated group evidenced by higher neutrophil, CD4, CD8, IFN‐γ, TNF and IL‐5 in combined treatment group compared to other groups	Wang *et al*.^88^

## Clinical studies combining radiotherapy with immune checkpoint blockade

### Thoracic cancer

There is emerging indirect clinical evidence that radiotherapy may enhance the efficacy of ICPB, and indeed, the addition of checkpoint blockade to chemoradiotherapy is now part of routine clinical care in stage III NSCLC. In the phase I KEYNOTE‐001 trial in which patients with NSCLC received pembrolizumab, a post hoc analysis revealed that patients who previously underwent any type of radiotherapy had longer progression‐free survival (PFS) than those who had not received radiotherapy. Three patients who had previously received thoracic radiotherapy experienced treatment‐related toxicity, compared to one of those who had not.^116^ The phase III randomised controlled, double‐blinded PACIFIC trial compared durvalumab (anti‐PD‐L1) to placebo in patients with stage III NSCLC who were progression‐free after receiving two or many cycles of platinum‐based chemoradiotherapy, increasing the median PFS from 5.6 to 16.8 months with the addition of durvalumab. Response rates in the durvalumab group were higher than the placebo group (28.4% vs. 16.0%; *P* < 0.001), and the median time to death or distant metastasis was also longer in patients receiving durvalumab (23.2 vs. 14.6 months).^117^ This is now considered standard of care for this patient group. Whilst demonstrating the feasibility and safety of radiotherapy plus ICPB, the additive effect of radiotherapy (or chemotherapy for that matter) is hard to ascertain here because of the absence of a durvalumab‐alone arm.

Whilst radiation‐induced abscopal effects have historically been reported at an extremely low frequency in the clinic, their incidence has anecdotally increased in patients receiving ICPB who have coincidentally received radiotherapy. In a patient with stage IV lung cancer failing to respond to chemotherapy, ipilimumab with local 6‐MV photon and a coplane 5‐field intensity‐modulated radiotherapy (30 Gy over five fractions over 10 days) resulted in a dramatic treatment response not only in the target areas, but also in distal sites. Post‐treatment biopsy showed an increase in tumor‐infiltrating lymphocytes.^118^ Another case report of metastatic NSCLC patient receiving high‐dose localised stereotactic body radiotherapy (3 × 6 Gy) with nivolumab given during and after radiotherapy revealed a complete radiological and metabolic response in treated tumor. Interestingly, a lymph node metastasis that was not irradiated also demonstrated a complete response, suggesting radiotherapy and nivolumab induced abscopal effects.^36^ Whilst dramatic abscopal effects remain clinically uncommon, they are reported with increased frequency in the context of ICPB. However, the specific tumor and radiotherapy conditions that may increase the likelihood of an abscopal response have not yet been identified.

## Optimal scheduling of radio‐immunotherapy

Whilst the potential of radiotherapy as an enhancer of response to ICPB has been demonstrated in several animal and human studies, the optimal scheduling of radiotherapy and ICPB remains unclear. To obtain the full potential of this combination, should ICPB be administered before, concurrent with, or after radiotherapy? The most accepted sequence and mechanism are to deliver radiotherapy before ICPB as, in cold tumors, radiotherapy could theoretically facilitate antigen and neoantigen release activating DCs and tumor‐specific T cells. However, some *in vivo* studies have shown that delivering radiotherapy after or concurrent with ICPB was superior to pre‐treatment (Table 2). The underlying mechanism for efficacy of later scheduling remains unclear. One possible explanation is that delivery of radiotherapy after or concurrent with ICPB occurs in the context of pre‐blockade of inhibitory receptor expression; hence, cellular infiltrates after radiotherapy all express inhibitory receptors, which may be harder to achieve with delivery of radiotherapy before ICPB.

**Table 2:** Selected clinical studies of scheduling between radiotherapy and ICPB

Cancer	Radiotherapy dose	Sequence	Outcomes	Study
Prostate cancer	8 Gy (single fraction)	Radiotherapy before ipilimumab	Clinical antitumor activities with disease control in a proportion of patients and generally controllable safety profile	Slovin *et al*.^142^
Melanoma brain metastasis	30–37 Gy (10–13 fractions)	Ipilimumab before radiotherapy vs. ipilimumab after radiotherapy	Overall survival was 18.4 months for patients taking ipilimumab after radiotherapy vs. 8.1 months patients receiving ipilimumab before radiotherapy	Silk *et al*.^143^
Melanoma	21 Gy	Comparison among three time points	Patients received radiotherapy during or before ICPB had better overall survival than radiotherapy after ICPB	Kiess *et al*.^122^
Melanoma brain metastasis	NA	Comparison between ipilimumab before and after radiotherapy	No significant differences in overall survival	Knisely *et al*.^144^
Metastatic melanoma	8–30 Gy (1–10 fractions) and one patient received 48 Gy	Concurrent therapy of radiotherapy with anti‐PD‐1 compared to sequential treatments	Concurrent administration of radiotherapy with anti‐PD‐1 had higher response rates than patients receiving sequential treatment	Liniker *et al*.^145^

There have been few *in vivo* studies of radio‐immunotherapy scheduling. In a model of colon carcinoma, the treatment with a single dose of αPD‐L1 mAb on day 1 or 5 of fractionated radiotherapy (10 Gy in 5 fractions) achieved long‐term survival of 60% and 57% of mice, respectively. However, giving αPD‐L1 mAb on day 7 after the completion of radiotherapy did not add to radiotherapy alone.^119^ Another study by Young and colleagues,^120^ using a mouse model of colorectal carcinoma, showed that anti‐CTLA‐4 was highly effective when given before 20 Gy, citing depletion of Tregs by anti‐CTLA‐4; however, anti‐OX‐40 antibodies were most effective 1 day after radiotherapy, within the active antigen presentation period. These studies, and the knowledge that each checkpoint inhibitor has a different mechanism of action and will interact uniquely with radiotherapy, underline the importance of careful study and emphasise that radiotherapy and checkpoint blockade will not be ‘one size fits all’.

Human studies will be required to resolve these questions clinically, and appropriate scheduling and sequencing may differ between cancer types, radiation doses and ICPB strategies. Nevertheless, because there are so many potential variables to be tested, careful *in vivo* studies should precede clinical trials in order to narrow the number of testable hypotheses. To date, a number of human case series also provide information, which can guide future clinical trial design. In 75 melanoma patients with a total of 566 brain metastases, given a median of 20 Gy SRS and anti‐PD‐1/anti‐CTLA‐4, concurrent immunotherapy and SRS led to a greater median per cent reduction in lesion volume than non‐concurrent therapy.^121^ Another series reviewed the safety and efficacy of combined SRS [median dose of 21 Gy (15–24 Gy)] with ipilimumab in 46 patients with melanoma brain metastases; overall survival and regional recurrence outcomes were better for patients who received SRS during or before, rather than after ipilimumab.^122^ In a phase I clinical trial of 22 patients with advanced melanoma, giving ipilimumab after hypofractionated radiotherapy to a single tumor lesion also led to partial responses in the non‐irradiated tumor, with a co‐clinical murine model demonstrating the best efficacy with triple therapy including anti‐PD‐L1.^123^ From another series of 88 consecutive patients with advanced melanoma, those receiving ipilimumab before radiotherapy (≥ 5 Gy) had longer response duration in irradiated tumors than those receiving ipilimumab after radiotherapy (75% vs. 45% control at 12 months).^124^ Indeed, finding a universal optimal time point for all clinical scenarios may not be realistic, and to date, neither preclinical nor clinical studies demonstrate a uniform best approach. Thus, the optimisation of timing in each clinical situation may be required to achieve the best synergistic effects of ICPB and radiotherapy.

## Biomarkers predicting response to radio‐immunotherapy combinations

Even if radio‐immunotherapy combinations that benefit patients are identified, it is unlikely that benefits will be uniform. Biomarkers that predict response will inform patients and clinicians, and may help clarify mechanisms of benefit. Each of tumor characteristics, immune infiltrate characteristics and changes in either tumor or immune cells after radiotherapy or ICPB may be potential biomarkers of clinical response to radio‐immunotherapy.

In some cancers, most notably NSCLC, the increased tumor expression of PD‐L1 predicts clinical responses to anti‐PD‐L1/anti‐PD‐1 monoclonal antibodies.^125^, ^126^ However, it is unclear how this relationship holds in the context of adding radiotherapy. A small retrospective cohort study in patients with oesophageal SCC and NSCLC reported that the increased PD‐L1 expression over the course of preoperative chemoradiotherapy was correlated with poor prognosis and shorter overall survival.^127^, ^128^ Other studies showed that initial high CD8^+^ T‐cell and PD‐L1 expression in tumor is associated with better therapeutic outcomes to radiotherapy in human papilloma virus‐induced cancers,^129^ oropharyngeal SCC^128^ and NSCLC.^62^

With respect to the immune milieu rather than tumor cells, some studies have reported MDSC and eosinophils as biomarkers for improved responses to cancer therapy such as in hepatocellular carcinoma.^130^ Cytokine production after radiotherapy may also predict therapeutic outcomes. For instance, in patients with solid tumors treated with radiotherapy, higher circulating IL‐6 and IL‐8 levels during the treatment predict for improved survival in head and neck cancer and rectal cancer.^131^, ^132^

Recently, the expression of the cGAS‐STING IFN type I synthesis pathway prior to starting treatment has been suggested as a biomarker to determine patients who will obtain durable responses from radiotherapy and ICPB combination in colorectal cancer.^133^, ^134^ In another cohort, increased HIF‐1α and vascular endothelial growth factor‐A anticipated poor responses to radiotherapy because of induction of Treg and MDSC migration to the TME.^135^ Overall, although these biomarkers may provide some indication of the likelihood of response, they have low specificity, and more research is warranted to validate candidate biomarkers, to broaden our understanding of the impacts of radiotherapy on the immune system – and thus optimise combinations of radiotherapy and ICPB.

## Conclusions and future directions

Radiotherapy is a fundamental part of cancer treatment. Numerous preclinical and clinical studies have shown the efficacy of combined radiotherapy with ICPB, with a sound biological rationale for synergy. Promising preclinical results have resulted in ongoing clinical trials. However, as demonstrated, radiotherapy may be a double‐edged sword; not only does it induce activation and infiltration of T cells to the tumor bed, but it can also trigger migration of immunosuppressive cells (e.g. MDSCs, M2 macrophage, Tregs) into the tumor and upregulate inhibitory ligands and receptors (PD‐L1, CTLA‐4, TIM‐3). Exploring the optimal doses, fractions and schedules for radiotherapy and ICPB may identify opportunities to modulate intratumoral immunosuppression and optimise combination therapy. Moreover, future studies may also consider depletion of immunosuppressive cell populations in addition to ICPB to abrogate negative signalling.

Translation of preclinical studies into successful clinical trials presents another challenge. The ultimate goal of preclinical studies is to accurately model the biological responses and toxicities of drugs in animals, in order to improve the chance of benefit, and reduce the risks, of human studies. Mice and humans have both differences and similarities with respect to the immune system and drug metabolism. Complete tumor regression observed in mice may not necessarily be mirrored in humans. Thus, it is important to determine the appropriate fraction and dose of radiotherapy, target selection, field size, the suitable logistical combinations of radiotherapy and immunotherapy, and the best biomarkers of response, in order to successfully translate the strategy into the clinic.

## Author Contributions

**Synat Keam:** Writing‐original draft. **Suki Gill:** Writing‐review & editing. **Martin Ebert:** Writing‐review & editing. **Alistair M Cook:** Writing‐original draft. **Anna K Nowak:** Writing‐review & editing.

## Conflict of Interest

The authors declare no conflict of interest.
